# Psilocybin with psychological support improves emotional face recognition in treatment-resistant depression

**DOI:** 10.1007/s00213-017-4754-y

**Published:** 2017-10-30

**Authors:** J. B. Stroud, T. P. Freeman, R. Leech, C. Hindocha, W. Lawn, D.J. Nutt, H.V Curran, R. L. Carhart-Harris

**Affiliations:** 1 Psychedelic Research Group, Neuropsychopharmacology Unit, Centre for Academic Psychiatry, Department of Medicine, Imperial College London, London, UK; 2 Clinical Psychopharmacology Unit, University College London, London, UK; 3 National Addiction Centre, Institute of Psychiatry, Psychology and Neuroscience, King’s College London, London, UK; 4 Computational, Cognitive and Clinical Neuroscience Laboratory, Department of Medicine, Imperial College London, London, UK

**Keywords:** Psilocybin, Emotional face recognition, Treatment-resistant depression, Anhedonia

## Abstract

**Rationale:**

Depressed patients robustly exhibit affective biases in emotional processing which are altered by SSRIs and predict clinical outcome.

**Objectives:**

The objective of this study is to investigate whether psilocybin, recently shown to rapidly improve mood in treatment-resistant depression (TRD), alters patients’ emotional processing biases.

**Methods:**

Seventeen patients with treatment-resistant depression completed a dynamic emotional face recognition task at baseline and 1 month later after two doses of psilocybin with psychological support. Sixteen controls completed the emotional recognition task over the same time frame but did not receive psilocybin.

**Results:**

We found evidence for a group × time interaction on speed of emotion recognition (*p* = .035). At baseline, patients were slower at recognising facial emotions compared with controls (*p* < .001). After psilocybin, this difference was remediated (*p* = .208). Emotion recognition was faster at follow-up compared with baseline in patients (*p =* .004, *d* = .876) but not controls (*p* = .263, *d* = .302). In patients, this change was significantly correlated with a reduction in anhedonia over the same time period (*r* = .640, *p* = .010).

**Conclusions:**

Psilocybin with psychological support appears to improve processing of emotional faces in treatment-resistant depression, and this correlates with reduced anhedonia. Placebo-controlled studies are warranted to follow up these preliminary findings.

**Electronic supplementary material:**

The online version of this article (10.1007/s00213-017-4754-y) contains supplementary material, which is available to authorized users.

## Introduction

Depressed patients exhibit negative affective biases in processing static emotional face stimuli, and these are thought to contribute to their low mood (Harmer et al. 2009). Selective serotonin or noradrenaline reuptake inhibitors (SSRIs, SNRIs), such as citalopram and reboxetine, respectively, have been shown to remediate these negative affective biases in depressed patients (Harmer et al. 2003a; Harmer et al. 2009). Also, in healthy volunteers these drugs produce a bias towards happy faces, both acutely and after 7 days’ administration (Harmer et al. 2003b; Norbury et al. 2009). These neuropsychological changes are thought to underlie the clinical effects of SSRIs and SNRIs (Warren et al. 2015) and are predictive of clinical outcome (Tranter et al. 2009; Shiroma et al. 2014).

In the real world, we process dynamic emotional expressions, as opposed to the static faces referred to above (Platt et al. 2010; Gepner et al. 2001; Robins et al. 2009). In an effort to increase the ecological validity of emotional face recognition, some researchers have developed dynamically changing facial expression tasks (Joormann and Gotlib 2006; Platt et al. 2010). As with static faces, negative affective biases have been observed in depressed patients with dynamic faces (Münkler et al. 2015) and these abnormalities in dynamic facial recognition are not evident in those who have responded to antidepressant medication (Anderson et al. 2011).

Psilocybin is a mixed serotonin receptor agonist that has displayed promising antidepressant potential (Carhart-Harris et al. 2012a, 2016). In healthy volunteers, it acutely impaired recognition of negative but not positive or neutral faces in the ‘Mind in the Eyes Task’ (Kometer et al. 2012) and increased psychological and brain responses to positive autobiographical memories (Carhart-Harris et al. 2012b). Revising negative cognitive and emotional biases in depression may be a viable mechanism through which psilocybin acts to reduce depressive symptoms (Harmer et al. 2017). However, to our knowledge, no study has examined whether psilocybin can produce longer-term changes in emotional face processing in people with depression, and whether these are associated with changes in depressive symptomology.

It is currently unclear why serotonergic drugs would act on specific emotions over others and whether psilocybin’s effects would differ from findings with SSRIs. Indeed, it has been proposed that 5-hydroxytryptamine (5-HT) agonists, such as psilocybin and SSRIs act via dissociable mechanisms—perhaps due to the former having a preferential action at 5-HT2A receptors and the latter, 5-HT1A receptors (Carhart-Harris and Goodwin 2017; Carhart-Harris and Nutt 2017).

In this study, we used a dynamically changing facial expression task (DEER-T, Platt et al. 2010) to investigate the impact of a psilocybin-based treatment on emotional processing biases in depressed patients. These outcomes were compared with test-retest data in a separate healthy control group. Patients took part in a recently reported pilot study of psilocybin (Carhart-Harris et al. 2016) and completed the task at pre-treatment baseline and 1 week after treatment. Controls did not take part in this trial but completed the task over a consistent time period. Our primary hypothesis was that the depressed patients’ would have impaired facial processing at baseline compared with controls and treatment with psilocybin would remediate this difference, as evidenced by a group × time interaction for reaction time data. We also predicted that this remediation effect would correlate with changes in depressive symptoms.

## Method

### Participants

Patients with treatment-resistant depression took part in a pilot study of psilocybin-based treatment. Findings have been reported regarding a subset (*n* = 12) of these patients (Carhart-Harris et al. 2016). Here, we present data from this initial sample plus an additional five patients, all of whom completed the DEER-T. Those with unipolar depression of at least moderate severity (17+ on the 21 item HAM-D) and who had not benefited from previous treatment with two antidepressant medications taken for at least 6 weeks were included. Seventeen of the 20 patients in the psilocybin depression study completed the DEER-T, and their data are reported here. For the purposes of this study, we additionally tested 16 healthy control participants twice over the same time period. Inclusion criteria for controls were (1) normal or corrected to normal vision, (2) able to complete computer tasks, (3) aged 18–60, and (4) fluent in English. Exclusion criteria were (1) a clinically diagnosed psychiatric illness and (2) a clinically diagnosed learning difficulty.

### Procedure

Full details of the pilot study procedures have been reported elsewhere (Carhart-Harris et al. 2016). Patients were screened at the Imperial Clinical Research Facility (ICRF). This involved informed consent, mental and physical health background documentation, the MINI-5 interview, physical examination, blood tests, ECG, urine test for drugs of abuse and pregnancy and an alcohol breathalyser. At this point, they also completed baseline measures including the 16-item Quick Inventory of Depressive Symptoms (QIDS-16, Rush et al. 2003), the Snaith-Hamilton Pleasure Scale (SHAPS, Snaith et al. 1995) and the Dynamic Emotional Expression Recognition Task (DEER-T, Platt et al. 2010).

Eligible patients attended two dosing sessions, separated by 1 week. In the first session, patients received 10 mg of psilocybin and in the second, they received 25 mg. The low-dose session was planned a priori as a safety session prior to a full-dose treatment session. This was included to minimise the likelihood of an adverse reaction during the high-dose session by familiarising patients with the study procedures and some of the subjective effects of psilocybin. The time point chosen to assess the efficacy of psilocybin was 1 week after the high-dose session in order to allow comparison with previous studies on ketamine in treatment-resistant depression (e.g. Zarate et al. 2006).

Psychological support comprised of the following, which can be summarised as *preparation*, (acute) *support* and *integration*: (1) *Preparation*: Prior to dosing, patients underwent an extensive preparatory session with their assigned psychiatrist and clinical psychologist or counsellor. This involved discussing their personal history (and depression), the subjective effects of psilocybin and an imitation of features of the dosing session, such as listening to a portion of the session music whilst wearing eyeshades. (2) (Acute) *support*: The dosing sessions took place in a pre-decorated room (e.g. low lighting, fabric drapes, flowers on bedside table) with two psychiatrists present. A non-directive, supportive approach was adopted, with the aim of allowing the patient an uninterrupted experience (Griffiths et al. 2016). Regular check-ins (asking the patient how they were feeling) occurred approximately every hour. (3) *Integration*: Patients met with their therapy pair 1 day after the high-dose session and were invited to talk through their experience. Compassionate listening and occasional interpretations were practised.

One week after the second treatment session (approximately 1 month after baseline), patients again returned to the ICRF for further integration work and to complete the DEER-T, the QIDS-16 and SHAPS. Controls did not take part in this trial but completed the task over a similar time period (1 month between ‘pre’ and ‘post’ time points). This was an opportunistic sample from an open-label trial and therefore the sample size was not based on a formal power calculation. Despite this, the sample size is similar (*n* = 17) to that used by Kometer et al. (2012).

### Dynamic Emotional Expression Recognition Task

Emotional processing was assessed with the DEER-T, which is described in detail elsewhere (Platt et al. 2010). Static colour photographs of six male and six female Caucasian actors were taken from the NimStim Face Stimulus Set (Tottenham et al. 2009). These were morphed to create six dynamic emotional stimuli expressing happiness, neutrality, sadness, anger, disgust, or fear. Stimuli morphed from neutral to a full display of an emotion over 3000 ms. There were six response buttons, one for each of the emotions. Participants were asked to press the key corresponding to the emotion becoming displayed as quickly and accurately as possible. The primary variable of interest is the reaction time on correct trials.

### Questionnaires

At baseline and 1 week after the high (25 mg) psilocybin dose, participants also completed the QIDS-16 (Rush et al. 2003) to index depression and the SHAPS (Snaith et al. 1995) to index lack of enjoyment of usually pleasurable activities. Controls completed the QIDS-16 on the same session as the DEER-T (pre and post) over the same time period.

### Statistical analysis

Group differences in age were analysed using an independent sample *t* test. Changes in SHAPS scores in patients were analysed using a paired sample *t* test (pre, post). A 2 × 2 repeated measures ANOVA was conducted on QIDS-16 scores with time as the within-subject factor and group as the between-subject factor. Reaction time, accuracy, sensitivity (Pr) and response bias (Br) data on the DEER-T were analysed using a 2 × 6 × 2 mixed ANOVA with time (pre and post) and emotion type (happy, neutral, sad, angry, disgusted and fearful) as within-subject factors and group (depressed patients and healthy controls) as the between-subject factor, followed up by Bonferroni-corrected *t* tests to explore any interactions. Cohen’s *d* (Cohen 1988) was calculated using the formula described by Morris and DeShon (2002). Missing data were handled using listwise deletion.

A two-high threshold model was used in order to calculate participants’ sensitivities (Pr) and response biases (Br) in recognising certain emotions (Snodgrass and Corwin 1988). The two-high threshold measures (Pr and Br) were used as non-parametric alternatives to signal detection measures (d’ and C) because accuracy data were not normally distributed. Sensitivity (Pr) reflects the probability, under conditions of uncertainty, that a certain emotional stimulus crosses a recognition threshold (Kamboj et al. 2012; Hindocha et al. 2014). Thus, the more sensitive one is to changes between emotions, the larger the value of Pr. Pr is calculated by subtracting the proportion of false alarms (p(FA): faces incorrectly identified as a certain emotion) from the proportion of hits (p(HIT): faces correctly identified as a certain emotion). Response bias (Br) is calculated by dividing the proportion of false alarms by one minus Pr (p(FA)/(1-Pr)). A larger Br value represents a more liberal approach and a lower value indicates a more conservative approach towards recognising a certain emotion.

## Results

### Demographics and depressive symptoms (Table 1)

Controls (*n* = 11 males, *n* = 5 females) had no psychiatric illness and were significantly younger (*p* = .002) with a mean age of 32 (*SD* = 10.40) than the patient group (*n* = 11 males, *n* = 6 females) with a mean age of 44.94 (*SD* = 11.51). All patients had previously used at least two antidepressant medications, seven were currently using psychiatric medication and withdrew or reduced these prior to dosing, all but two patients had received some form of counselling or psychotherapy with six receiving CBT and five patients also had a previous diagnosis of another psychiatric condition. Patient demographics for a subset of this sample (*n* = 12) have been described in more detail elsewhere (Carhart-Harris et al. 2016).

**Table 1:** Depressive symptoms for patients and controls at the ‘pre’ and ‘post’ time points

	Patients		Controls	
	Pre	Post	Pre	Post
QIDS-16	18.88 (2.23)	7.65 (5.34)	4.13 (3.90)	4.13 (3.32)
SHAPS	6.82 (4.02)	1.47 (2.43)	–	–

In patients, SHAPS scores were significantly reduced at the ‘post’ treatment time point as compared to the ‘pre’ treatment time point, indicating an improved capacity for experiencing pleasure following psilocybin-based treatment (*F* (1, 16) = 37.14, *p* < .001, *η*_*p*_^2^ = .699). For QIDS-16 scores, there was a significant interaction effect of time and group (*F* (1, 31) = 68.18, *p* < .001, *η*_*p*_^2^ = .687). There were main effects of time (*F* (1, 31) = 68.18, *p* < .001, *η*_*p*_^2^ = .687) and group (*F* (1, 31) = 61.63, *p* < .001, *η*_*p*_^2^ = .665). Bonferroni-corrected pair-wise *t* tests (*α* = .025) revealed that the QIDS-16 scores for patients were significantly lower (*p* < .001) at the ‘post’ time point (*M* = 7.65, *SD* = 5.34) as compared to the ‘pre’ time point (*M* = 18.88, *SD* = 2.23), reflecting a reduction in depressive symptoms following psilocybin-based treatment. No significant difference in QIDS-16 scores was observed for controls between the ‘pre’ and ‘post’ time points (*p* = 1.00). These findings have been presented elsewhere (Carhart-Harris et al. 2016).

### Dynamic Emotional Expression Recognition Task

#### Reaction time

There were significant interaction effects between time and group (*F* (1, 28) = 4.91 *p* = .035, *η* ^2^ = .149) time and emotion (*F* (5, 140) = 2.67, *p* = .025, *η*_*p*_^2^ = .087) and emotion and group (*F* (5, 140) = 2.56, *p* = .030, *η*_*p*_^2^ = .084). There were also significant main effects of time (*F* (1, 28) = 12.10, *p* = .002, *η*_*p*_^2^ = .302), group (*F* (1, 28) = 11.02, *p* = .003, *η*_*p*_^2^ = .282) and emotion (*F* (5, 140) = 78.23, *p* < .001, *η*_*p*_^2^ = .736).

**Fig. 1:**
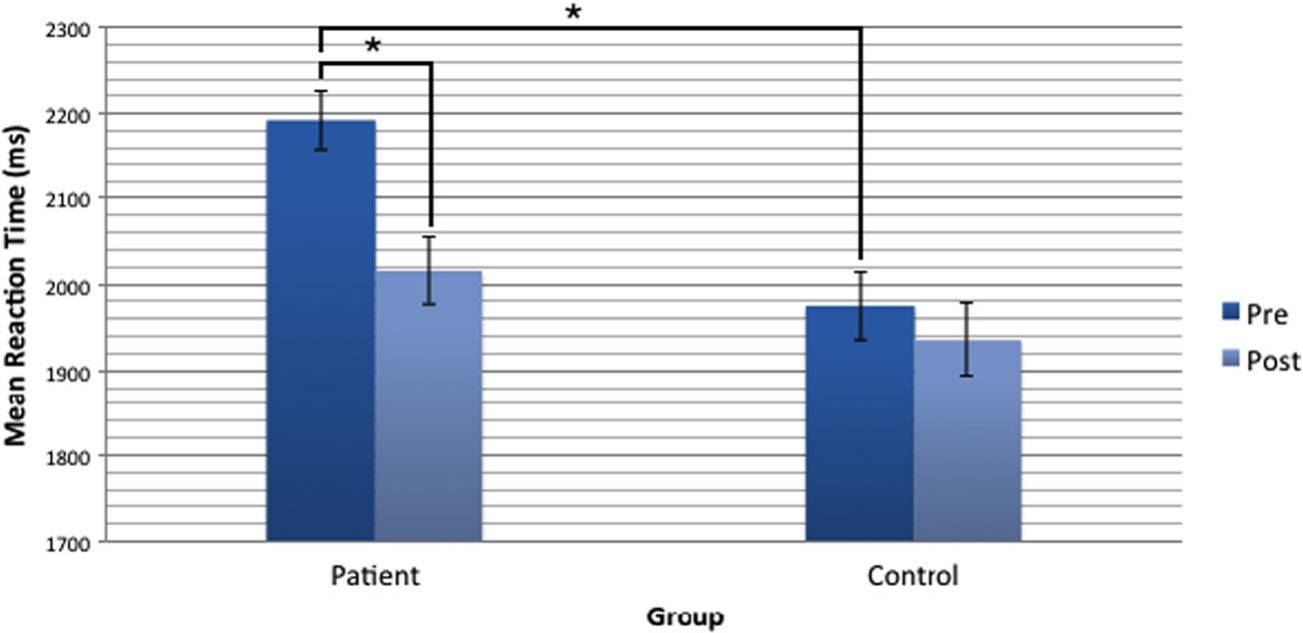
Mean reaction time to correctly respond to all emotions on the DEER-T task in patients and controls. Patients were significantly faster at the ‘post’ time point as compared with the ‘pre’ time point (*p* = .004). Reaction time for controls was not significantly different between the two time points (*p* = .263). Controls were significantly faster than patients at the ‘pre’ time point (*p* < .001) but at the ‘post’ time point there was no significant difference between the two groups (*p* = .208). Error bars are ± 1 SE and * denotes a *p* value < .0125

Bonferroni-corrected pair-wise *t* tests (*α* = .0125) to examine the time by group interaction revealed that the reaction time across all emotions for patients was significantly faster (*p* = .004, *d* = .876) at the ‘post’ time point (*M* = 2015.48, *SE* = 38.84) as compared with the ‘pre’ time point (*M* = 2192.58, *SE* = 34.38), whereas the reaction time for controls was not significantly different (*p* = .263, *d* = .302) across the two time points (Fig. 1). Moreover, controls were significantly faster than patients at the ‘pre’ time point (*p* < .001) but at the ‘post’ time point, there was no significant difference between the two groups (*p* = .208).

Bonferroni-corrected pair-wise *t* tests (*α* = .008) to examine the time by emotion interaction revealed that the reaction time in both groups combined for angry (*M* = 2006.86, *SE* = 36.26) faces at the ‘post’ time point was significantly faster (*p* = .001) as compared with angry (*M* = 2147.25, *SE* = 43.31) faces at the ‘pre’ time point. There were no significant differences in reaction time across both groups between ‘pre’ and ‘post’ for the other emotions.

Bonferroni-corrected pair-wise *t* tests (*α* = .008) to examine the emotion by group interaction revealed that reaction times were significantly faster in the control group for neutral (*p* = .003), angry (*p* < .001), disgusted (*p* = .001) and fearful (*p* = .001) faces as compared with the patient group. Means and standard errors are reported in Online Resource 3. There was no significant interaction of emotion, group and time (*F* (5, 140) = .993, *p* = .424, *η*_*p*_^2^ = .034).

#### Accuracy

There was a significant interaction effect of emotion and group (*F* (5, 27) = 3.75, *p* = .009, *η*_*p*_^2^ = .108). There were also significant main effects of time (*F* (1, 31) = 12.5, *p* = .001, *η*_*p*_^2^ = .287) and emotion (*F* (3.63, 112) = 35.44, *p* < .001, *η*_*p*_^2^ = .533) but not group (*p* = .119). Bonferroni-corrected (*α* = .008) independent sample *t* tests to examine the emotion by group interaction showed that across both time points, controls were significantly more accurate (*p* < .001) when responding to angry faces (*M* = .8930, *SE* = .018) as compared with patients (*M* = .7479, *SE* = .03). There were no significant differences between groups on the other emotions. There were no significant interactions of time and group (*p* = .124), time and emotion (*p* = .057), or emotion, group and time (*p =* .240).

#### Discrimination

There was a significant interaction effect of emotion and group (*F* (3.72, 115) = 2.95, *p* = .026, *η*_*p*_^2^ = .087). There were also significant main effects of time (*F* (1, 31) = 9.97, *p* = .004, *η*_*p*_^2^ = .243) and emotion (*F* (3.72, 115) = 34.69, *p* < .001, *η*_*p*_^2^ = .528) but not group (*p* = .197). Bonferroni-corrected (*α* = .008) independent sample *t* tests to examine the emotion by group interaction showed that sensitivity (Pr) across both time points was significantly higher for angry faces (*p* = .002) in controls (*M* = .831, *SE* = .168) as compared with patients (*M* = .717, *SE* = .032). There were no significant interactions of time and emotion (*F* (3.36, 104) = 1.77, *p* = .152, *η*_*p*_^2^ = .054) or time and group (*F* (1, 31) = 2.18, *p* = .150, *η*_*p*_^2^ = .066) or time, group and emotion (*F* (3.36, 104) = 1.66, *p* = .149, *η*_*p*_^2^ = .051).

#### Response bias

There were significant interaction effects of time and emotion (*F* (3.26, 101) = 4.45, *p* = .004, *η*_*p*_^2^ = .126) and emotion and group (*F* (3.54, 110) = 7.04, *p* < .001, *η*_*p*_^2^ = .185). There were also significant main effects of time (*F* (1, 31) = 4.86, *p* = .035, *η*_*p*_^2^ = .136), emotion (*F* (3.54, 110) = 27.57, *p* < .001, *η*_*p*_^2^ = .471) and group (*F* (1, 31) = 18.71, *p* < .001, *η*_*p*_^2^ = .376). Bonferroni-corrected (*α* = .008) paired sample *t* tests to examine the time by emotion interaction showed that across both groups, there was a significantly more liberal response bias (*p* = .001) towards neutral faces at the ‘post’ time point (*M* = .255, *SE* = .033) as compared to the ‘pre’ time point (*M* = .148, *SE* = .025). Response bias for other emotions across both groups did not significantly differ from the ‘pre’ time point to the ‘post’ time point. Bonferroni-corrected (*α* = .008) independent sample *t* tests to examine the emotion by group interaction showed that across both time points, there was a significantly more liberal response bias towards angry faces (*p* < .001) in controls (*M* = .35, *SE* = .036) as compared with patients (*M* = .128, *SE* = .017). No significant group differences in response bias (Br) were found for the other emotions across both time points. There were no significant interaction effects of time and group (*p* = .711) or emotion, group and time (*p =* .867*).*

#### Correlations

**Fig. 2:**
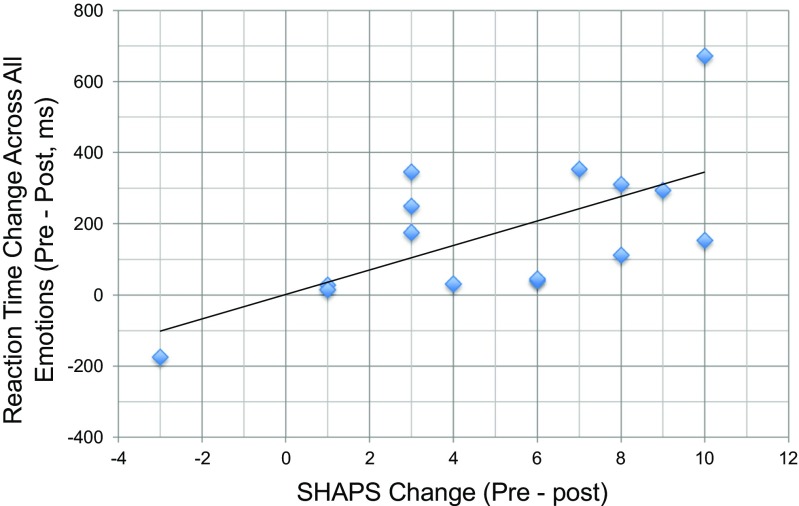
Change in reaction time to all emotions correlated with change in SHAPS score in depressed patients receiving psilocybin-based treatment (*r* = .640, *p* = .010). The larger the decrease in reaction time to detect emotional faces, the larger the reduction in anhedonia

Given that patients showed a reduction in reaction time for all emotions, and improved symptomology on the QIDS-16 and SHAPS, correlations were conducted between change scores on these variables in the patient group. A significant positive correlation was observed between change in reaction time across all emotions and change in SHAPS score in patients (*r* = .640, *p* = .010, Fig. 2). This indicates that faster identification of emotional expressions was associated with reduced anhedonia in patients. This relationship appeared to be relatively selective to this particular depressive symptom, as QIDS-16 score change was not significantly correlated with reaction time change in patients (*r* = − .073, *p* = .796). Because age significantly differed between patients and controls, we explored correlations between age and reaction time to respond to all emotions. We found no evidence for an association in patients (*r* = − .299, *p* = .278) or controls (*r* = .375, *p* = .169). There was also no evidence for an association between *change* in reaction time to respond to all emotions and age in patients (*r* = .106, *p* = .707) or controls (*r* = .196, *p* = .484) or in both groups combined (*r* = − .099, *p* = .603).

## Discussion

Patients treated with psilocybin with psychological support for treatment-resistant depression and a group of healthy controls receiving no treatment carried out a dynamic emotional faces task at two time points, separated by approximately 1 month. Prior to treatment with psilocybin, depressed patients in this trial were shown to have a global deficit in processing emotional faces as compared with healthy controls, as reflected in longer reaction times to identify all emotion types. Post-treatment, this between-group difference was remediated—with patients performing as well as controls. We observed a reaction time improvement post-treatment for all emotion types in depressed patients. In the control group, who were not given psilocybin, no retest improvement was observed. This suggests the improvement in the depressed group is *not* due to a learning effect but rather, may be related to their treatment.

We observed a significant positive correlation between faster RT to all emotions and improvements in anhedonia scores post-treatment. Anhedonia (lack of pleasure or interest in normally rewarding activities) is a key component of depression—and yet it is relatively unresponsive to standard antidepressant treatments (Lally et al. 2014). Both psilocybin with psychological support (Carhart-Harris et al. 2016) and ketamine (Lally et al. 2014) appear to facilitate an immediate improvement in anhedonia in depression. It has been suggested that ketamine’s fast-acting antidepressant effect may be due to large and immediate neurocognitive changes (Warren et al. 2015; Deakin et al. 2012; Abel et al. 2003) that subsequently allow fast relearning of patients’ social environments (Pringle et al. 2013). Our observed correlation suggests that improvements in facial processing relate to contemporaneous improvements in symptoms of anhedonia, in line with the neurocognitive model of antidepressant action (Warren et al. 2015). No correlations were observed between facial processing performance and the study’s primary measure of depressive symptoms (QIDS-16), suggesting that the post-treatment changes in emotional processing are selective to the symptom of anhedonia.

It has been recently proposed that psilocybin activates an emotion-*releasing* pathway mediated via the 5-HT2A receptor whilst SSRIs act on an emotion-*moderating* pathway mediated by postsynaptic 5-HT1AR signalling (Carhart-Harris and Goodwin 2017, Carhart-Harris and Nutt 2017). SSRIs have been associated with emotional moderation or ‘blunting’ (Price et al. 2009), whilst 15 out of 20 patients in the current trial endorsed a theme antithetical to this, labelled ‘emotional reconnection’ and ‘acceptance’ (Watts et al. 2017). Speculatively, the improved processing of *all* emotions, including negative ones, seen here post-treatment with psilocybin could be interpreted as consistent with emotional *reconnection* (Watts et al. 2017; Carhart-Harris et al. 2017). Indeed, recent research using the same sample as the current study with an additional two patients (*n* = 19) has shown increased amygdala response to fearful faces as compared with neutral faces after psilocybin (Roseman et al. 2017, Neuropsychopharmacology, under review). Reconnecting with one’s emotions and environment could account for the inter-related improvements in anhedonia and emotional processing post-psilocybin.

Focusing on group differences irrespective of treatment, an emotion × group interaction effect was observed, where across both time points combined, controls were faster at responding to neutral, angry, disgusted and fearful faces as compared with the depressed group. This finding is broadly consistent with the findings of Dalili et al.’s (2015) meta-analysis of 22 emotional face processing studies, which concluded that there was a global deficit for all basic emotions (anger, disgust, fear, happiness and surprise) except sadness in those with depression (Dalili et al. 2015). A key limitation here is that no three-way interaction was observed; thus, any interpretation of emotion-specific deficits across group and time should be treated with caution. Moreover, although age did not correlate with task performance, the two groups differed in age.

We also observed a time by emotion interaction effect, where reaction time for angry faces in both groups combined was significantly faster at the ‘post’ time point. This may reflect a differential learning effect on angry faces in this task that is insensitive to group, as we did not see a significant interaction with group on this measure.

There was a significant emotion by group interaction effect where across both time points combined, controls were more accurate at identifying angry faces. This was also reflected in a higher discrimination index (Pr) and a move to the ideal response bias (Br = 0.5) for angry faces in controls across both time points combined as compared to patients. This may reflect the fact that the emotional processing deficit in patients towards angry faces is resilient to change post-psilocybin. Although it should be noted that in this task, the faces become increasingly intense over time and thus easier to answer correctly. Indeed, following previous use of this task (Platt et al. 2010) reaction time should be the principal measurement from which to make conclusions.

In addition to emotional processing aptitude, performance on facial recognition tasks also involves a variety of cognitive functions such as selective attention, working memory and visual perception (Stuhrmann et al. 2011). Depressed patients are known to show cognitive impairments including both mnemonic and executive impairments (Austin et al. 2001) as well as perceptual deficits (Bschor et al. 2004; Bubl et al. 2010). Bearing this in mind, whether the depressed patients in our study showed a specific improvement in emotional processing or a more general cognitive improvement (e.g. executive function) or even perceptual (e.g. contrast sensitivity) ability remains to be resolved. To do so would require the inclusion of other behavioural paradigms in the trial, probing different aspects of affective and cognitive processing. In the future, it would be valuable to include other cognitive and perceptual tasks, in order to test whether psilocybin-treatment has a selective influence on emotional face processing or a more generalised remediating effect on affective and cognitive processing.

Strengths of the present study include its focus on a novel pharmacological mechanism associated with rapid improvement in treatment-resistant depression, pre/post-assessments in patients and controls, and a previously validated emotional processing task based on dynamic face morphing. There are several limitations to this study however, namely (1) controls were significantly younger than the patients and age has been found to affect emotional face recognition (Leime et al. 2013), (2) controls did not undergo a placebo treatment as they would in a randomised control trial (RCT), (3) additional cognitive tasks were not administered, (4) the sample size was modest, (5) SHAPS was not collected in controls and (6) the treatment group had psilocybin and psychological support whilst the control group had neither. There is some evidence that extended psychological intervention (16 sessions) may influence processing of facial affect in depression (Fu et al. 2008). Future studies should aim to determine whether psychological support and psilocybin have additive or synergistic effects on depressive symptoms and emotion recognition (Moss et al. 2016). Bearing in mind the ethical concerns of a psilocybin-only group in people with depression, this may be best achieved by comparing a minimum level of support with increasing doses of psychological intervention. In the future, studies could also incorporate an eye-tracking paradigm, as an intriguing explanation for reduced performance on emotional face recognition tasks may be aversion of gaze away from emotional stimuli. Many of these limitations were standard for an open-label pilot study such as it was, and can be addressed properly in future RCTs.

In conclusion, this study found generalised improvements in emotional face processing after psilocybin for treatment-resistant major depression that correlated with reduced anhedonia. Further studies are required to help determine whether the observed improvements are specific to emotion processing or more generalised, i.e. including improvements in perception and/or cognition; if the former, it will be important to address whether improvements in emotional processing are an integral component of the antidepressant mechanisms of this novel treatment modality (Carhart-Harris and Goodwin 2017).

## Electronic supplementary material

ESM 1 (DOCX 126 kb)
