# Supplementary material for: Psilocybin with psychological support improves emotional face recognition in treatment-resistant depression
Source: Psychopharmacology (Berl). 2017 Oct 30;235(2):459–66. doi: 10.1007/s00213-017-4754-y (PMC5813058; doi:10.1007/s00213-017-4754-y)
Supplement: Supplementary file 1 — (DOCX 126 kb) [file 213_2017_4754_MOESM1_ESM.docx]

Supplementary Materials

**Title: Psilocybin with psychological support improves emotional face recognition in treatment resistant depression**

Authors: Stroud, J.B.^1,2^, Freeman, T.P.^2,3^, Leech, R.^4^, Hindocha, C.^2^, Lawn, W.^2^, Nutt, D.^1^ , Curran, V.^2^ and Carhart-Harris, R. L.^1^.

^1^ Psychedelic Research Group, Neuropsychopharmacology Unit, Centre for Academic Psychiatry, Department of Medicine, Imperial College London,

^2^ Clinical Psychopharmacology Unit, University College London, London, UK

^3^ National Addiction Centre, Institute of Psychiatry, Psychology and Neuroscience, King’s College London, London, UK

^4^ Computational, Cognitive and Clinical Neuroscience Laboratory, Department of Medicine, Imperial College London, London, UK

Corresponding author: Jack Stroud

Email: jack.stroud.16@ucl.ac.uk

Phone: 020 7679 1897


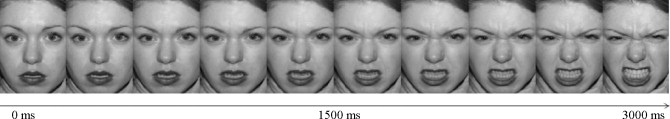


Online Resource 1: Trial sequence in the Dynamic Emotional Expression Recognition Task (DEER-T). Participants are required to identify the correct emotion (anger shown in this trial) as the face morphs from neutral to full emotion.

|  | Patient | | Control | |
| --- | --- | --- | --- | --- |
| Reaction Time (ms) | Pre | Post | Pre | Post |
| Happy | 1796.81 (49.30) | 1576.47 (58.83) | 1614.77 (49.30) | 1636.86 (58.83) |
| Neutral | 2233.83 (56.50) | 1974.10 (61.18) | 1992.42 (56.50) | 1840.39 (61.18) |
| Sad | 2229.02 (48.99) | 2165.45 (48.57) | 2124.61 (48.99) | 2122.96 (48.57) |
| Angry | 2283.71 (50.55) | 2110.59 (44.21) | 2010.80 (50.55) | 1903.12 (44.21) |
| Disgusted | 2325.07 (47.69) | 2145.34 (57.17) | 2061.40 (47.69) | 2069.80 (57.17) |
| Fearful | 2287.05 (49.30) | 2120.93 (60.56) | 2044.77 (49.30) | 2039.90 (60.56) |

Online Resource 2: Mean reaction times (standard error) for each emotion on the DEER-T task in patients and controls at 'pre' and 'post' time points.

|  | **Happy** | **Neutral** | **Sad** | **Angry** | **Disgusted** | **Fearful** |
| --- | --- | --- | --- | --- | --- | --- |
| **Patient** | 1686.64 (41.25) | 2103.97 (46.74) | 2197.23 (40.36) | 2197.15 (39.37) | 2235.21 (36.90) | 2203.99 (47.71) |
| **Control** | 1625.82 (41.25) | 1916.41 (42) | 2123.79 (40.36) | 1957.00 (39.26) | 2065.60 (40.57) | 2042.33 (43.30) |
|  |  |  |  |  |  |  |

Online Resource 3: Mean reaction times (standard error) for each emotion on the DEER-T task in patients and controls combined across both the ‘pre’ and ‘post’ time point.
